# Medical Department of University of Buffalo

**Published:** 1853-12

**Authors:** 


*Medical Department of University of Buffalo.—Summer Course of Recitations and Lectures for* 1851.—Conducted by Drs. Thomas F. Rochester and Sanford B. Hunt, commencing on the first Monday in April, and ending on the last Saturday in October. During the month of August the course will be suspended.

Examinations on Anatomy, Surgery, Chemistry, Materia Medica, Obstetrics, and Theory and Practice of Medicine, will be held daily at 11, A. M.

Lectures will occasionally be given on subjects connected with the above departments.

Drs. White and Hamilton have kindly consented to give a few lectures on subjects connected with their respective chairs.

It is believed that such a course as that above announced is called for by the true interests of the student; and the best efforts of the instructors will be given to his thorough preparation for the duties of his profession.

Terms: $50 for the course, payable in advance.

Buffalo, November, 1853.

In thus giving publicity to this notice we wish to add a word as to its objects. The necessity of preparatory schools of medicine is now universally felt and acknowledged, and this extension of the curriculum is intended to supply that necessity in Western New York. Since the issue of the foregoing circular a College Clinic has been established in the edifice of the school, and students attending this course will have the important advantages to be derived from it. This clinic will be full and comprehensive, continuing throughout the year, and is entirely under the control of the College Faculty. It is established in adddition to the already excellent clinic to which all the students of the regular term are admitted, and we trust that these efforts on the part of the Faculty to give new life and energy to medical teaching in Buffalo, will meet the approbation of the profession.

All the different branches will be thus taught practically. Anatomy will be studied in the dissection room, chemistry in the laboratory, while therapeutical and surgical art will be illustrated by the presence of disease.

